# Gastrocnemius activation deficits and running biomechanics after anterior cruciate ligament reconstruction: the missing link?

**DOI:** 10.3389/fspor.2025.1594247

**Published:** 2025-05-09

**Authors:** Florian Forelli, Ayrton Moiroux-Sahraoui, Jean Mazeas, Anais Pengue Koyi, Mouna Labib, Adrien Cerrito

**Affiliations:** ^1^ Haute-Ecole Arc Santé, HES-SO University of Applied Sciences and Arts Western Switzerland, Delémont, Switzerland; ^2^ Orthopaedic Surgery Department, Clinic of Domont, Ramsay Healthcare, @OrthoLab, Domont, France; ^3^ Société Française des Masseurs—Kinésithérapeutes du Sport Lab, Pierrefitte sur Seine, France; ^4^ Orthosport Rehab Center, Domont, France

**Keywords:** anterior cruciate ligament reconstruction, gastrocnemius activation, neuromuscular deficits, running, biomechanic analysis

## Abstract

**Background:**

Return to running (RTR) after anterior cruciate ligament reconstruction (ACL-R) remains challenging due to persistent neuromuscular deficits. This study examines gastrocnemius activation and running biomechanics in ACL-R patients.

**Methods:**

Fifteen ACL-R patients and fifteen healthy controls were assessed using surface electromyography during isometric testing and treadmill running (10 km.h^−1^). Spatiotemporal parameters, including ground contact time, flight time, step width, cadence, stride length, and vertical stiffness, were analyzed.

**Results:**

ACL-R patients exhibited lower gastrocnemius activation during isometric testing (medial: 48.5% vs. 55.9% MVIC, *p* = 0.01; lateral: 42.1% vs. 47.5% MVIC, *p* = 0.03) and during running (medial: 45.2% vs. 53.1% MVIC, *p* < 0.01; lateral: 39.7% vs. 44.8% MVIC, *p* = 0.04). They also demonstrated altered running biomechanics, including longer ground contact time (0.29 vs. 0.26 s, *p* = 0.02, *d* = − 0.5), shorter stride length (1.32 vs. 1.41 m, *p* = 0.03, d = 0.9), reduced vertical stiffness (21.8 vs. 25.6 kN.m^−1^, *p* = 0.03, *d* = 0.5), and slightly increased step width (0.14 vs. 0.13 m, *p* = 0.05, *d* = 0.4). A significant negative correlation was observed between medial gastrocnemius activation during running and ground contact time (rs = −0.56, *p* = 0.02, ES = −0.6). Lateral gastrocnemius activation was positively correlated with stride length (r*s* = 0.49, *p* = 0.03, ES = 0.5), and medial gastrocnemius activation showed a moderate positive correlation with vertical stiffness (rs = 0.52, *p* = 0.04, ES = 0.5). Cadence did not show a statistically significant correlation with either medial or lateral gastrocnemius activation (rs = 0.36, *p* = 0.08, ES = 0.4 and rs = 0.45, *p* = 0.09, ES = 0.4, respectively).

**Conclusion:**

Gastrocnemius dysfunction persists after ACL-R, affecting running mechanics. These findings suggest that current rehabilitation protocols may need to incorporate plantar flexor training to optimize running mechanics post-ACL-R.

## Introduction

Anterior cruciate ligament reconstruction (ACL-R) is a common surgical intervention aimed at restoring knee stability following its rupture. Despite surgical advancements, the rehabilitation process remains complex, particularly in terms of ensuring a safe and effective return to running (RTR) (1). Running is a fundamental component of many sports, and its resumption is often considered a critical milestone in post-operative recovery (2). However, current RTR criteria focus predominantly on clinical knee assessments, quadriceps and hamstring muscle strength, and functional tests (3–6). While these criteria provide valuable insight into knee function, they largely overlook the contribution of the gastrocnemii muscles, which play a crucial role in propulsion, stability, and force transmission during running (7–9).

Studies have highlighted that biomechanical asymmetries persist in lower limbs post-ACL-R, often lasting beyond six months and contributing to altered neuromuscular control (10, 11). Such asymmetries can lead to compensatory strategies, which not only impair running efficiency but also increase the risk of reinjury and osteoarthritis development (12–14). Although surface electromyographic (sEMG) analysis has been employed to study muscle activation patterns in ACL-R patients, limited research has specifically examined the gastrocnemii muscle's involvement in post-operative running mechanics (9, 15, 16). Given that the gastrocnemii play a pivotal role in ankle plantarflexion and force modulation, a deeper understanding of its activation post-ACL-R is essential.

Among the plantar flexor muscles, the gastrocnemius plays a particularly relevant role in running due to its biarticular nature, spanning both the knee and ankle joints. This anatomical configuration allows it to contribute not only to ankle plantarflexion but also to knee flexion, making it a key muscle for propulsion and dynamic stability during the stance phase of running (7). In contrast, the soleus is a monoarticular muscle that acts solely at the ankle and is predominantly composed of type I muscle fibers, contributing more to postural control and endurance-based activities (8). Moreover, the gastrocnemius is more active during faster and more explosive movements, and its recruitment is phase-dependent with a peak during late stance and push-off, which are critical for forward propulsion (17). From a methodological standpoint, sEMG is more reliable and accessible for the gastrocnemius due to its superficial location, whereas the soleus, being a deep muscle, is difficult to assess accurately without invasive techniques (18). Therefore, gastrocnemius represents a more feasible and functionally relevant target for understanding after ACL-R neuromuscular adaptations and their impact on running biomechanics.

Furthermore, muscle function assessments in ACL-R rehabilitation predominantly emphasize quadriceps and hamstring strength, often overlooking plantar flexor function (19, 20). This research gap raises critical questions about the appropriateness of current RTR guidelines, particularly regarding whether patients adequately regain neuromuscular control in the gastrocnemius muscles before resuming high-impact activities. Addressing this gap may contribute to improved rehabilitation protocols and safer return-to-sport decision-making.

The primary aim of this study is to investigate the electromyographic activity and strength of the gastrocnemius muscles in ACL-R patients during RTR and compare them with healthy controls. Specifically, this study seeks to analyze gastrocnemius muscle activation patterns in ACL-R patients relative to non-injured individuals using sEMG (15, 21). It also examines differences in spatiotemporal running parameters, such as stride length, cadence, and contact time, between ACL-R patients and healthy subjects (11, 22). Potential correlations between gastrocnemius muscle activity and these running parameters are determined to assess their role in neuromuscular adaptations following ACL-R (9, 16, 23).

By addressing these objectives, this study aims to enhance understanding of the gastrocnemii muscle's function in post-ACL-R locomotion, refine RTR criteria, and inform rehabilitation strategies that optimize functional recovery and reduce reinjury risk.

We hypothesized that individuals following ACL_R would exhibit increased gastrocnemius activation and altered running biomechanics compared to healthy controls, and that these neuromuscular deficits would be significantly associated with key spatiotemporal running parameters.

## Methods

### Study design

This study is a controlled, cross-sectional observational study designed to compare the electromyographic activity and strength of the gastrocnemius muscles in ACL-R patients during RTR with that of healthy control participants. Data collection was conducted in a controlled laboratory environment using sEMG and kinematic assessments to evaluate neuromuscular function and running biomechanics (24).

Ethical approval for the study was obtained from the Institutional Review Board, ensuring compliance with ethical research guidelines. All participants provided written informed consent before participation in accordance with the Declaration of Helsinki (World Medical Association, 2013). Participants were informed of the study objectives, procedures, potential risks, and their right to withdraw at any time without consequences.

### Sample size calculation

*Post hoc* sample size estimation was conducted using G*Power 3.1 to determine the minimum number of participants required per group to detect a large effect size (*d* = 0.8) with a power of 0.80 and an alpha level of 0.05. The analysis indicated that a minimum of 26 participants per group would be necessary.

### Participants

A total of 30 participants were recruited, including 15 ACL-R patients (199.2 days ± 28.1), and 15 healthy control subjects matched for age, sex, Tegner and Marx Activity Scale. The ACL-R group consisted of individuals who underwent surgical reconstruction performed by three different surgeons using the same protocol at least 3 months prior and were cleared for RTR based on clinical criteria.

### Inclusion criteria

Participants had to be between 18 and 40 years of age, have undergone unilateral ACL reconstruction using a hamstring graft, and be involved in sports requiring running prior to their injury. All participants have been operated on by three orthopedic surgeons following the same standardized surgical procedure. Completion of a standard rehabilitation protocol (2, 25–27) and limb symmetry index (LSI) of at least 70% for quadriceps and hamstrings strength on an 60°s^−1^ isokinetic assessment was required, together with a numeric pain score below 2 and no joint effusion (Swipe Test <1+) (3, 28). Additionally, they needed at least 70% of contralateral performance on the Single Hop Test and the Single-Leg Vertical Jump Test. They also had to be free of pain or swelling at the time of testing.

### Exclusion criteria

Participants with bilateral ACL injuries, additional ligamentous or meniscal injuries requiring surgical repair, lower limb fractures in the past year, or neurological conditions affecting movement were excluded. Those with ongoing pain, functional limitations, or a history of previous ACL reconstruction in the contralateral knee were also ineligible. Additionally, individuals undergoing current rehabilitation for other musculoskeletal injuries or with contraindications for running, such as cardiovascular conditions, were excluded (29).

### Assessment protocol

Participants performed a standardized assessment protocol beginning with a general warm-up consisting of five minutes of light cycling and dynamic stretching. sEMG electrodes (BTS Bioengineering, Garbagnate Milanese, Italy, 1,000 Hz) were placed on the medial and lateral gastrocnemius muscles following Surface Electromyography for the Non-Invasive Assessment of Muscles (SENIAM) guidelines, ensuring optimal electrode placement at the muscle belly while avoiding the myotendinous junction (18). Skin preparation included shaving, abrasion, and alcohol cleansing to reduce impedance and oil deposits. Electrodes were secured with adhesive tape to prevent movement artifacts during testing.

Triceps surae strength and gastrocnemius maximum voluntary isometric contraction (MVIC) were assessed using an isokinetic dynamometer (Biodex System 4, Biodex Medical Systems, New York, USA, 2,000 Hz) with participants seated on the dynamometer chair, the backrest slightly tilted. The participants were seated with the hip flexed at 110° and the knee bent between 70° and 80°, ensuring that the tibia remained parallel to the ground (30–32). The ankle was fixed at 0° dorsiflexion, with the fibular malleolus aligned with the axis of rotation of the dynamometer. The foot was securely attached to the footplate, and participants were stabilized with a waist strap and two shoulder straps. They held onto the shoulder straps to minimize extraneous movements. The dynamometer arm was adjusted to align precisely with the ankle joint, and participants performed three 5-s MVIC trials, separated by 30-s rest intervals. Verbal encouragement and real-time feedback were provided to ensure maximal effort. Prior to analysis, the raw EMG signals were band-pass filtered between 20 and 450 Hz using a fourth-order zero-lag Butterworth filter to remove movement artifacts and high-frequency noise. The signals were then full-wave rectified and smoothed using a root mean square (RMS) algorithm with a 50 ms sliding window. The RMS amplitude was extracted and used as the reference value for further analysis. This processing pipeline allowed for reliable quantification of muscle activation during both isometric and dynamic running conditions.

Running trials were conducted on a motorized treadmill OptoGait system (Microgate, Bolzano, Italy,1,000 Hz), a validated optical measurement system for gait analysis. The OptoGait system consists of an LED transmitter and receiver bar positioned on either side of a treadmill to capture spatiotemporal parameters with high precision. All participants performed the running assessment at a fixed speed of 10 km/h for a duration of three minutes (33). Spatiotemporal parameters, including stride length, step width, flight time, cadence, vertical stiffness and ground contact time, were recorded with OptoGait system (Microgate, Bolzano, Italy,1,000 Hz).

Although kinetic analysis and musculoskeletal modeling would provide additional biomechanical insights, this study focused on surface electromyography and spatiotemporal parameters to explore neuromuscular deficits under more accessible and clinically translatable conditions. This choice aligns with the study's objective to inform real-world rehabilitation protocols.

### Statistical analysis

Descriptive statistics were computed for all demographic and clinical variables, including mean and standard deviations for continuous variables and frequency distributions for categorical variables. Normality of data was assessed using the Shapiro–Wilk test.

For between-group comparisons, independent t-tests were used for normally distributed variables, while Mann–Whitney *U* tests were applied to non-normally distributed data.

Correlation analyses were conducted using Pearson's correlation coefficient for parametric data and Spearman's rank correlation for non-parametric data to explore relationships between gastrocnemius activation, spatiotemporal running parameters, and limb symmetry indices. We performed a stepwise multiple regression analysis to identify significant predictors of neuromuscular performance and return-to-running success.

A significance level of *p* < 0.05 was used for all analyses. Confidence intervals were reported at 95%, and all statistical analyses were carried out using SPSS version 26.0 (IBM Corp., Armonk, New York, USA). To control multiple comparisons, Bonferroni corrections were applied where necessary.

## Results

### Participant characteristics

The ACL-R group had an average age of 27.3 ± 4.20 years, while the control group had an average age of 26.8 ± 4.00 years (*p* *=* *0.67*). The mean height was 1.75 ± 0.06 m for ACL-R patients and 1.76 ± 0.07 m for controls (*p* *=* *0.72*). Body weight averaged 72.50 ± 6.30 kg in the ACL-R group and 73.0 ± 6.50 kg in the control group (*p* *=* *0.81*). No significant differences were observed in these parameters between the groups (Table 1).

**Table 1:** Participant characteristics.

Data	ACL-R (*n* = 15)	Control (*n* = 15)	*p*-value
	Mea*n* ± SD	Mean ± SD	
Age (year)	27.30 ± 4.20	26.80 ± 4.00	0.67
Height (m)	1.75 ± 0.06	1.76 ± 0.07	0.72
Weight (kg)	72.50 ± 6.30	73.00 ± 6.50	0.81
Women (*n*, %)	5 (33%)	6 (40%)	0.78
Tegner Score	6.5 ± 1.3	6.6 ± 1.7	0.95
Marx Score	6.7 ± 1.4	7.8 ± 2.8	0.53

ACL-R, anterior cruciate ligament reconstruction; SD, standard deviation; m, meter; kg, kilogram.

### Triceps surae muscle strength

The maximum isometric strength of the triceps surae was significantly lower in the ACL-R group, with a mean force of 105.60 ± 11.90 Nm compared to 120.30 ± 12.40 Nm in controls (*p* *=* *0.02*).

### Gastrocnemius muscle activity during isometric testing

During isometric testing (Table 2), the medial gastrocnemius activation was significantly lower in ACL-R patients (48.5 ± 7.9% MVIC) compared to controls (55.9 ± 8.3% MVIC, *p* *=* *0.01*). The lateral gastrocnemius also showed reduced activation, with ACL-R patients reaching 42.1 ± 6.4% MVIC, whereas controls exhibited 47.5 ± 6.9% MVIC (*p* *=* *0.03*). These findings suggest altered motor patterns in the gastrocnemii following ACL reconstruction.

**Table 2:** Gastrocnemius muscle activity during isokinetic testing.

Data	ACL-R (*n* = 15)	Control (*n* = 15)	*p*-value	ES
	Mean ± SD	Mean ± SD		
Medial gastrocnemius (%MVIC)	48.50 ± 7.90	55.90 ± 8.30	0.01	−0.9
Lateral gastrocnemius (%MVIC)	42.10 ± 6.40	47.50 ± 6.90	0.03	−0.2

ACL-R, anterior cruciate ligament reconstruction; MVIC, maximum voluntary isometric contraction; SD, standard deviation.

### Gastrocnemius muscle activity during running

During running (Table 3), the medial gastrocnemius activation was significantly lower in ACL-R patients, with a mean activation of 45.20 ± 8.10% MVIC compared to 53.10 ± 7.80% MVIC in controls (*p* *<* *0.01*). Similarly, the lateral gastrocnemius was less activated in ACL-R patients (39.70 ± 6.50% MVIC) than in controls (44.80 ± 7.20% MVIC, *p* *=* *0.04)*.

**Table 3:** Gastrocnemius muscle activity during running.

Data	ACL-R (*n* = 15)	Controls (*n* = 15)	*p*-value	ES
	Mean ± SD	Mean ± SD		
Medial gastrocnemius (%MVIC)	45.20 ± 8.10	53.10 ± 7.80	<0.01	0.8
Lateral gastrocnemius (%MVIC)	39.70 ± 6.50	44.80 ± 7.20	0.04	0.5

ACL-R, anterior cruciate ligament reconstruction; MVIC, maximum voluntary isometric contraction; SD, standard deviation.

### Spatiotemporal running parameters

All reported *p*-values in the correlation analyses (Table 4) reflect Bonferroni-adjusted values to account for multiple comparisons (adjusted *α* = 0.01). Ground contact time was significantly longer in ACL-R patients, averaging 0.29 ± 0.01 s, compared to 0.26 ± 0.01 s in controls (*p* *=* *0.02*). Stride length was reduced in ACL-R patients, with an average of 1.32 ± 0.05 m vs. 1.410 ± 0.04 m in controls (*p* *=* *0.03*). Vertical stiffness was also significantly lower in ACL-R patients, at 21.80 ± 3.500 kN·m^−^^1^ compared to 25.60 ± 3.90 kN·m^−^^1^ in controls (*p* *=* *0.03*). The cadence of ACL-R patients was higher, with a mean of 166.50 ± 6.40 steps/min, whereas controls reached 150.20 ± 5.90 steps.min^−1^ (*p* *=* *0.07*), though this difference was not statistically significant. Flight time was reduced in ACL-R patients, averaging 0.11 ± 0.01 s, compared to 0.12 ± 0.01 s in the control group (*p* *=* *0.04*), indicating a lower ability to generate effective propulsion. Step width was slightly increased in ACL-R patients, measuring 0.14 ± 0.01 m, while controls had an average step width of 0.13 ± 0.01 m (*p* *=* *0.05*) (Table 4).

**Table 4:** Spatiotemporal running parameters.

Data	ACL-R (*n* = 15)	Controls (*n* = 15)	*p*-value	Cohen's *d*
	Mean ± SD	Mean ± SD		
Ground contact time (s)	0.29 ± 0.01	0.26 ± 0.01	0.02	−0.5
Stride length (m)	1.32 ± 0.05	1.41 ± 0.04	0.03	0.9
Vertical stiffness (kN·m ^−^^1^ )	21.80 ± 3.50	25.60 ± 3.90	0.03	0.5
Cadence (steps.min ^−1^ )	166.50 ± 6.40	150.20 ± 5.90	0.07	0.9
Flight time (s)	0.11 ± 0.01	0.12 ± 0.01	0.04	0.2
Step width (m)	0.14 ± 0.02	0.13 ± 0.01	0.05	0.4

*p*-values are Bonferroni-corrected for multiple comparisons*.* ACL-R, anterior cruciate ligament reconstruction; MVIC, maximum voluntary isometric contraction; SD, standard deviation; s, second; m, meter; kN, kilonewton.

### Correlations between muscle activity and spatiotemporal running parameters

A significant negative correlation was observed between medial gastrocnemius activation during running and ground contact time (rs = −0.56, *p* = 0.02, ES = −0.6). Lateral gastrocnemius activation was positively correlated with stride length (rs = 0.49, *p* = 0.03, ES = 0.5). Vertical stiffness exhibited a moderate positive correlation with medial gastrocnemius activation (rs = 0.52, *p* = 0.04, ES = 0.5). Cadence did not show a significant correlation with either medial or lateral gastrocnemius activation (rs = 0.36, *p* = 0.08, ES = 0.4 and rs = 0.45, *p* = 0.09, ES = 0.4, respectively).

## Discussion

The primary aim of this study was to evaluate neuromuscular function in ACL-R patients by analyzing gastrocnemius muscle activation and its relationship with spatiotemporal running parameters. The findings confirm that ACL-R patients exhibit persistent deficits in muscle activation and running mechanics after completion of a standard rehabilitation protocol compared to healthy controls. The reduced gastrocnemii activation observed both during isometric testing and running suggests impaired neuromuscular patterns, which may contribute to biomechanical inefficiencies. Furthermore, the correlation analyses reveal significant associations between muscle activity and key running parameters, highlighting the impact of gastrocnemii function on dynamic performance. These results align with previous studies showing prolonged neuromuscular deficits following ACL reconstruction, emphasizing the need for rehabilitation strategies targeting plantar flexor function (16, 19).

The persistence of neuromuscular impairments in ACL-R patients raises concerns about the effectiveness of current rehabilitation protocols in fully restoring lower limb function (6, 34, 35). Although traditional rehabilitation programs prioritize quadriceps and hamstring strengthening, the deficits observed in gastrocnemii activation suggest that the recovery of plantar flexor function is not adequately addressed. Given that the gastrocnemii contribute significantly to propulsion and shock absorption during running, reduced activation of these muscles may be associated with some of the biomechanical alterations observed in this study (7, 11).

During isometric testing, ACL-R patients demonstrated significantly lower activation of both medial and lateral gastrocnemius muscles compared to healthy controls. This decrease suggests deficits in motor unit recruitment and neuromuscular drive, likely influenced by altered proprioceptive feedback and central nervous system adaptations following ACL injury and its surgical management (36–39). The inability to effectively activate the gastrocnemii may result in decreased ankle plantarflexion force, which is crucial for stabilizing the knee joint and assisting in shock absorption (12, 40, 41).

The reduced gastrocnemius activation observed during running supports the possibility of ongoing neuromuscular alterations following ACL reconstruction. Lower activation levels may result in decreased force production during push-off, limiting forward propulsion and altering stride mechanics (16, 40). This decrease in muscle engagement can lead to a redistribution of mechanical demands, with increased reliance on proximal muscle groups such as the hamstrings and hip extensors to compensate for plantar flexor deficiencies (42–45). Such compensatory strategies may not fully restore optimal running biomechanics and could contribute to secondary musculoskeletal impairments, including patellofemoral pain syndrome or Achilles tendinopathy due to altered load distribution (46).

The alterations in spatiotemporal parameters observed in ACL-R patients provide further evidence of compromised running mechanics. The prolonged ground contact time suggests an inability to generate rapid force during the stance phase, possibly due to reduced plantar flexor strength and inefficient neuromuscular coordination (47). This adaptation may increase the metabolic cost of running and contribute to early fatigue, potentially limiting return-to-sport performance (22). The lengthened stance phase may also indicate a protective strategy aimed at reducing joint loading, yet such adaptations could compromise running efficiency and expose ACL-R patients to additional biomechanical stressors (12).

The reduction in stride length further indicates impaired propulsive capacity, likely reflecting the observed deficits in gastrocnemii activation. Since stride length is closely linked to running efficiency and overall performance, these findings suggest that ACL-R patients may struggle to achieve optimal gait patterns even after rehabilitation (12, 16, 43, 45). Furthermore, the significant increase in vertical stiffness supports the notion that ACL-R patients exhibit a reduced ability to store and return elastic energy, a key factor in efficient running mechanics (11, 23). This may reflect impairments in the stretch-shortening cycle of the gastrocnemii, further limiting its contribution to propulsion and force attenuation.

The slight increase in step width may represent a compensatory strategy aimed at improving postural stability during dynamic movements. Previous studies have indicated that ACL-R patients often adopt wider steps to enhance balance control and mitigate instability risks (43–46). However, this adaptation may come at the cost of increased mechanical demand on the lower limb and altered muscle coordination patterns. A wider base of support can alter lower limb joint kinetics and may lead to increased medial-lateral ground reaction forces, which could contribute to inefficient movement patterns and potentially higher injury risks in the long term (6, 48).

The correlation analyses provide further insight into the functional impact of gastrocnemius activation on running biomechanics. The significant negative correlation between medial gastrocnemius activation and ground contact time (rs = −0.56, *p* = 0.02, ES = −0.6) suggests that higher muscle activation is associated with a shorter stance phase, which could contribute to improved running efficiency (22). This finding indicates that deficits in gastrocnemii function may directly prolong stance duration, reducing the effectiveness of propulsion and increasing mechanical loading on the knee joint.

Similarly, the positive correlation between lateral gastrocnemius activation and stride length (rs = 0.49, *p* = 0.03, ES = 0.5) indicates that greater neuromuscular engagement may enhance forward propulsion, supporting the role of the gastrocnemii in optimizing gait mechanics (40). This suggests that insufficient activation of the lateral gastrocnemius may contribute to shorter strides, reinforcing the hypothesis that ACL-R patients adopt inefficient movement strategies due to persistent neuromuscular impairments.

The lateral head is more involved in horizontal propulsion, contributing significantly to forward movement during activities such as running and jumping (17). In contrast, the medial head is primarily responsible for postural stabilization, aiding in the maintenance of balance and control during standing and slow movements (49). This functional differentiation may explain why activation of the lateral gastrocnemius correlates with stride length, as its engagement directly influences propulsion dynamics (17, 49).

After ACL-R, patients often develop compensatory strategies to mitigate deficits in quadriceps strength and neuromuscular control. These adaptations may include increased reliance on the lateral gastrocnemius to assist in propulsion, while the medial head's involvement in propulsion diminishes, potentially due to its stabilizing role being compromised by altered neuromuscular patterns. Additionally, after ACL-R neuromuscular impairments and changes in lower limb alignment can disrupt the activation patterns of the gastrocnemius muscle, further reducing the medial head's contribution to gait mechanics.

While gastrocnemii was the focus of the present study, we acknowledge that the soleus also plays a critical role in ankle plantarflexion and contributes substantially to propulsion and vertical support during running. Its predominantly slow-twitch fiber composition and monoarticular structure make it particularly suited for endurance and postural control functions. However, due to its deep anatomical location beneath the gastrocnemius, surface EMG does not allow for reliable and selective recording of soleus activity without significant crosstalk or signal degradation (18). This methodological constraint led us to focus our analysis on the gastrocnemius, which is both functionally important and accessible using non-invasive sEMG techniques. We recognize this as a limitation and suggest that future studies consider using intramuscular EMG or advanced imaging methods to assess soleus activation in conjunction with gastrocnemius function. A more comprehensive understanding of the plantar flexor group may help further refine rehabilitation protocols after ACL reconstruction.

Vertical stiffness was also positively correlated with medial gastrocnemius activation (rs = 0.52, *p* = 0.04), highlighting the muscle's contribution to energy storage and return during dynamic movements. Reduced vertical stiffness has been linked to increased mechanical energy dissipation, which may lead to greater fatigue and decreased running economy (50–54). These findings suggest that restoring adequate gastrocnemii function is essential for optimizing the stretch-shortening cycle and improving running performance.

In contrast, cadence did not show significant correlations with gastrocnemius activation (rs = 0.36, *p* = 0.08 and rs = 0.45, *p* = 0.09, respectively) suggesting that stride frequency adjustments in ACL-R patients may be influenced by other neuromuscular adaptations rather than direct plantar flexor involvement (16, 55). This highlights the complexity of post-ACL-R running mechanics, where multiple factors, including proprioceptive deficits, quadriceps activation asymmetries, and altered motor control strategies, interact to shape movement patterns.

One possible explanation for the reduced gastrocnemius activation observed in ACL-R patients, despite having completed rehabilitation, may lie in persistent neuromuscular alterations. Several mechanisms could account for this, including persistent quadriceps-gastrocnemius co-contraction patterns that inhibit gastrocnemius recruitment (56), arthrogenic muscle inhibition linked to altered afferent input from the injured joint (57), and possible deconditioning or neuromuscular reorganization resulting from altered gait patterns early in recovery (19). Moreover, most rehabilitation programs focus primarily on quadriceps and hamstring recovery, potentially under-addressing plantar flexor function. These mechanisms could contribute to ongoing neuromuscular alterations despite apparent functional recovery.

These results underscore the critical role of gastrocnemii in determining running performance after ACL reconstruction. Persistent deficits in neuromuscular function may contribute to inefficient gait mechanics, prolonged ground contact times, and reduced propulsive capacity, ultimately affecting return-to-sport readiness. Future research should further investigate how targeted interventions can enhance gastrocnemii function and restore optimal neuromuscular coordination, potentially improving running efficiency and reducing reinjury risk in ACL-R patients. Additionally, longitudinal studies assessing the long-term consequences of these neuromuscular impairments will be crucial in refining rehabilitation strategies and optimizing functional recovery.

## Limits

This study has several limitations that should be considered when interpreting the findings. First, the relatively small sample size may limit the generalizability of the results. A larger cohort would improve statistical power and help confirm the observed correlations.

Second, our analysis was limited to surface electromyography and spatiotemporal parameters. We did not include kinematic or kinetic measurements such as joint angles, ground reaction forces, or joint moments, which restricts our ability to explore detailed joint mechanics or load distribution. While these measures require more complex setups, their integration—particularly through musculoskeletal modeling—could provide a deeper understanding of ACL strain, patellofemoral joint loading, and compensatory strategies during running (58, 59).

Third, we did not objectively measure the ankle range of motion. As a result, it remains unclear whether reduced gastrocnemius activation reflects neuromuscular inhibition or mechanical restrictions such as limited dorsiflexion. Future research should include a standardized range of motion assessments (e.g., weight-bearing lunge test) to clarify this distinction and better tailored rehabilitation strategies.

Fourth, the protocol was conducted at a fixed submaximal speed (10 km.h ^−1^ ) under non-fatiguing conditions. While this ensured consistency across participants, it may have masked endurance-related neuromuscular deficits that often emerge with fatigue. Including prolonged running trials or fatigue protocols in future studies could help reveal compensatory changes that affect return-to-sport readiness.

Finally, the cross-sectional design prevents any inference about causality between gastrocnemius dysfunction and altered biomechanics. Longitudinal studies tracking recovery and adaptation over time are needed to determine whether these deficits persist despite rehabilitation and whether targeted interventions can improve outcomes.

## Conclusion

This study identifies reduced gastrocnemii activation and altered spatiotemporal running parameters in ACL-R patients, which may reflect neuromuscular adaptations potentially linked to running performance and injury risk. The findings suggest that inadequate plantar flexor recruitment contributes to prolonged ground contact times, reduced stride length, and impaired propulsion, all of which may affect return-to-sport readiness. The observed correlations between muscle activation and biomechanical parameters further support the importance of neuromuscular control in optimizing running mechanics. Future studies should focus on longitudinal assessments and targeted rehabilitation strategies to restore gastrocnemius function and improve running efficiency in ACL-R patients.

## Data Availability

The original contributions presented in the study are included in the article/Supplementary Material, further inquiries can be directed to the corresponding author.

## References

[1] Van Cant J Pairot De Fontenay B Douaihy C Rambaud A. Characteristics of return to running programs following an anterior cruciate ligament reconstruction: a scoping review of 64 studies with clinical perspectives. Phys Ther Sport. (2022) 57:61–70. 10.1016/j.ptsp.2022.07.006 35921783

[2] Rambaud AJ Neri T Edouard P. Reconstruction, rehabilitation and return-to-sport Continuum after anterior cruciate ligament injury (ACLR3-Continuum): call for optimized programs. Ann Phys Rehabil Med*.* (2022) 65:101470. 10.1016/j.rehab.2020.101470 33333209

[3] Rambaud AJM Ardern CL Thoreux P Regnaux J-P Edouard P. Criteria for return to running after anterior cruciate ligament reconstruction: a scoping review. Br J Sports Med*.* (2018) 52:1437–44. 10.1136/bjsports-2017-098602 29720478

[4] Beynnon BD Vacek PM Newell MK Tourville TW Smith HC Shultz SJ The effects of level of competition, sport, and sex on the incidence of first-time noncontact anterior cruciate ligament injury. Am J Sports Med*.* (2014) 42:1806–12. 10.1177/0363546514540862 25016012 PMC6604059

[5] Grindem H Snyder-Mackler L Moksnes H Engebretsen L Risberg MA. Simple decision rules can reduce reinjury risk by 84% after ACL reconstruction: the Delaware-Oslo ACL cohort study. Br J Sports Med*.* (2016) 50:804–8. 10.1136/bjsports-2016-096031 27162233 PMC4912389

[6] Forelli F Le Coroller N Gaspar M Memain G Kakavas G Miraglia N Ecological and specific evidence-based safe return to play after anterior cruciate ligament reconstruction in soccer players: a new international paradigm. Int J Sports Phys Ther*.* (2023) 18(2):526–40. 10.26603/001c.73031 37020454 PMC10069338

[7] Neptune RR Wright IC Den Bogert AJV. A method for numerical simulation of single limb ground contact events: application to heel-toe running. Comput Methods Biomech Biomed Engin*.* (2000) 3:321–34. 10.1080/10255840008915275 11264857

[8] Lichtwark GA Wilson A.M. Interactions between the human gastrocnemius muscle and the Achilles tendon during incline, level and decline locomotion. J Exp Biol*.* (2006) 209:4379–88. 10.1242/jeb.02434 17050853

[9] Forelli F Mazeas J Pengue-Koyi A Kakavas G Hewett TE Rambaud A. Higher gastrocnemius muscle activity may impair running spatio-temporal parameters after anterior cruiciate ligament reconstruction: a pilot study. Sport Perf Sci. (2023):1–4.

[10] Schmitt LC Paterno MV Hewett TE. The impact of quadriceps femoris strength asymmetry on functional performance at return to sport following anterior cruciate ligament reconstruction. J Orthop Sports Phys. Ther*.* (2012) 42:750–9. 10.2519/jospt.2012.4194 22813542 PMC4157226

[11] Wellsandt E Failla MJ Snyder-Mackler L. Limb symmetry indexes can overestimate knee function after anterior cruciate ligament injury. J Orthop Sports Phys Ther*.* (2017) 47:334–8. 10.2519/jospt.2017.7285 28355978 PMC5483854

[12] Paterno MV Ford KR Myer GD Heyl R Hewett TE. Limb asymmetries in landing and jumping 2 years following anterior cruciate ligament reconstruction. Clin J Sport Med*.* (2007) 17:258–62. 10.1097/JSM.0b013e31804c77ea 17620778

[13] Noehren B Hamill J Davis I. Prospective evidence for a hip etiology in patellofemoral pain. Med Sci Sports Exerc*.* (2013) 45:1120–4. 10.1249/MSS.0b013e31828249d2 23274607

[14] Palmieri-Smith RM Thomas AC. A neuromuscular mechanism of posttraumatic osteoarthritis associated with ACL injury. Exerc Sport Sci Rev*.* (2009) 37:147–53, 10.1097/JES.0b013e3181aa6669 19550206

[15] Konishi Y Fukubayashi T Takeshita D. Mechanism of quadriceps femoris muscle weakness in patients with anterior cruciate ligament reconstruction. Scand J Med Sci Sports. (2002) 12:371–5. 10.1034/j.1600-0838.2002.01293.x 12453165

[16] Dingenen B Malfait B Nijs S Peers KHE Vereecken S Verschueren SMP Postural stability during single-leg stance: a preliminary evaluation of noncontact lower extremity injury risk. J Orthop Sports Phys Ther*.* (2016) 46:650–7, 10.2519/jospt.2016.6278 27374015

[17] Arndt AN Komi PV Brüggemann G-P Lukkariniemi J. Individual muscle contributions to the in vivo Achilles tendon force. Clin Biomech. (1998) 13:532–41. 10.1016/S0268-0033(98)00032-1 11415831

[18] Hermens HJ Freriks B Disselhorst-Klug C Rau G. Development of recommendations for SEMG sensors and sensor placement procedures. J Electromyogr Kinesiol. (2000) 10:361–74. 10.1016/S1050-6411(00)00027-4 11018445

[19] Palmieri-Smith RM Thomas AC Wojtys EM. Maximizing quadriceps strength after ACL reconstruction. Clin Sports Med*.* (2008) 27:405–24. 10.1016/j.csm.2008.02.001 18503875

[20] Lepley AS Gribble PA Thomas AC Tevald MA Sohn DH Pietrosimone BG. Quadriceps neural alterations in anterior cruciate ligament reconstructed patients: a 6-month longitudinal investigation. Scand J Med Sci Sports. (2015) 25:828–39. 10.1111/sms.12435 25693627

[21] Cui H Cao Z Wang S Zhang H Chen Z Wu X Surface electromyography characteristics of patients with anterior cruciate ligament injury in different rehabilitation phases. Front Physiol*.* (2023) 14:1116452. 10.3389/fphys.2023.1116452 37051018 PMC10083235

[22] Sigward SM Pollard CD Havens KL Powers CM. Influence of sex and maturation on knee mechanics during side-step cutting. Med Sci Sports Exerc*.* (2012) 44:1497–503, 10.1249/MSS.0b013e31824e8813 22330027 PMC3399059

[23] Buckthorpe M Roi GS. The time has come to incorporate a greater focus on rate of force development training in the sports injury rehabilitation process. Muscle Ligaments Tendons J*.* (2019) 07:435. 10.32098/mltj.03.2017.05 PMC5774916 29387636

[24] Merletti R Farina D. Surface Electromyography: Physiology, Engineering, and Applications. Hoboken, NJ: Wiley (2016).

[25] van Melick N van Cingel REH Brooijmans F Neeter C van Tienen T Hullegie W Evidence-based clinical practice update: practice guidelines for anterior cruciate ligament rehabilitation based on a systematic review and multidisciplinary consensus. Br J Sports Med. (2016) 50:1506–15. 10.1136/bjsports-2015-095898 27539507

[26] Kotsifaki R Korakakis V King E Barbosa O Maree D Pantouveris M Aspetar clinical practice guideline on rehabilitation after anterior cruciate ligament reconstruction. Br J Sports Med*.* (2023) 57:500–14. 10.1136/bjsports-2022-106158 36731908 PMC11785408

[27] Forelli F Sansonnet C Chiapolini S Mazeas J Vandebrouck A Duffiet P Optimizing return to play after anterior cruciate ligament reconstruction in soccer players: an evidence based approach. [Preprint]. (2020). 10.20944/preprints202012.0447.v1

[28] Rambaud AJM Semay B Samozino P Morin J-B Testa R Philippot R Criteria for return to sport after anterior cruciate ligament reconstruction with lower reinjury risk (CR’STAL study): protocol for a prospective observational study in France. BMJ Open. (2017) 7:e015087. 10.1136/bmjopen-2016-015087 28667211 PMC5734254

[29] Myer GD Paterno MV Ford KR Quatman CE Hewett TE. Rehabilitation after anterior cruciate ligament reconstruction: criteria-based progression through the return-to-sport phase. J Orthop Sports Phys Ther*.* (2006) 36:385–402. 10.2519/jospt.2006.2222 16776488

[30] Al-Uzri M O’Neill S Watson P Kelly C. Reliability of isokinetic dynamometry of the plantarflexors in knee flexion and extension. Physiother Pract Res*.* (2016) 38:49–57. 10.3233/PPR-160084

[31] Santy F-M Pernoud A Barrué-Belou S Fourchet F Bothorel H Samozino P. Reliability of isokinetic dynamometer for isometric assessment of ankle plantar flexor strength. Phys Ther Sport. (2025) 71:36–42. 10.1016/j.ptsp.2024.11.001 39626379

[32] Tuominen J Leppänen M Jarske H Pasanen K Vasankari T Parkkari J. Test−retest reliability of isokinetic ankle, knee and hip strength in physically active adults using biodex system 4 pro. Methods Protoc*.* (2023) 6:26. 10.3390/mps6020026 36961046 PMC10037623

[33] DeJong Lempke AF Audet AP Wasserman MG Melvin AC Soldes K Heithoff E Biomechanical differences and variability during sustained motorized treadmill running versus outdoor overground running using wearable sensors. J Biomech. (2025) 178:112443. 10.1016/j.jbiomech.2024.112443 39626380

[34] Biały M Wilczyński B Forelli F Hewett TE Gnat R. Functional deficits in non-elite soccer (football) players: a strength, balance, and movement quality assessment after anterior cruciate ligament reconstruction. Cureus. (2024) 16(12):e75846. 10.7759/cureus.75846 39822459 PMC11738117

[35] Moiroux–Sahraoui A Forelli F Mazeas J Rambaud AJ Bjerregaard A Riera J. Quadriceps activation after anterior cruciate ligament reconstruction: the early bird gets the worm! Int J Sports Phys Ther*.* (2024) 19(8):1044–51. 10.26603/001c.121423 39100933 PMC11297573

[36] Konishi Y Konishi H Fukubayashi T. Gamma loop dysfunction in quadriceps on the contralateral side in patients with ruptured ACL. Med Sci Sports Exerc*.* (2003) 35:897–900. 10.1249/01.MSS.0000069754.07541.D2 12783035

[37] Konishi Y Yoshii R Ingersoll CD. Gamma loop dysfunction as a possible neurophysiological mechanism of arthrogenic muscle inhibition: a narrative review of the literature. J Sport Rehabil*.* (2022) 31:736–41. 10.1123/jsr.2021-0232 35078149

[38] Grooms DR Page SJ Nichols-Larsen DS Chaudhari AMW White SE Onate JA. Neuroplasticity associated with anterior cruciate ligament reconstruction. J Orthop Sports Phys Ther*.* (2017) 47:180–9. 10.2519/jospt.2017.7003 27817301

[39] Needle AR Lepley AS Grooms DR. Central nervous system adaptation after ligamentous injury: a summary of theories, evidence, and clinical interpretation. Sports Med*.* (2017) 47:1271–88. 10.1007/s40279-016-0666-y 28005191

[40] Ferri-Caruana A Sendra-Pérez C Priego-Quesada JI. Gastrocnemius neuromuscular activation during standing explosive acceleration. Life Basel Switz*.* (2024) 14:1378. 10.3390/life14111378 PMC11595705 39598177

[41] Hof AL Van Zandwijk JP Bobbert MF. Mechanics of human triceps surae muscle in walking, running and jumping. Acta Physiol Scand*.* (2002) 174:17–30. 10.1046/j.1365-201x.2002.00917.x 11851593

[42] Gokeler A Benjaminse A Hewett TE Paterno MV Ford KR Otten E Feedback techniques to target functional deficits following anterior cruciate ligament reconstruction: implications for motor control and reduction of second injury risk. Sports Med Auckl NZ. (2013) 43:1065–74. 10.1007/s40279-013-0095-0 PMC4166506 24062274

[43] Erhart-Hledik JC Chu CR Asay JL Andriacchi TP. Gait mechanics 2 years after anterior cruciate ligament reconstruction are associated with Longer-term changes in patient-reported outcomes. J Orthop Res Off Publ Orthop Res Soc*.* (2017) 35:634–40. 10.1002/jor.23317 PMC5823008 27238273

[44] Butler RJ Minick KI Ferber R Underwood F. Gait mechanics after ACL reconstruction: implications for the early onset of knee osteoarthritis. Br J Sports Med*.* (2009) 43:366–70. 10.1136/bjsm.2008.052522 19042923

[45] Skvortsov D Kaurkin S Akhpashev A Altukhova A Troitskiy A Zagorodniy N. Gait analysis and knee kinematics in patients with anterior cruciate ligament rupture: before and after reconstruction. Appl Sci*.* (2020) 10:3378. 10.3390/app10103378

[46] Di Stasi S Myer GD Hewett TE. Neuromuscular training to target deficits associated with second anterior cruciate ligament injury. J Orthop Sports Phys Ther*.* (2013) 43:777; A11. 10.2519/jospt.2013.4693 24175599 PMC4163697

[47] Neptune RR Kautz SA Zajac FE. Contributions of the individual ankle plantar flexors to support, forward progression and swing initiation during walking. J Biomech*.* (2001) 34:1387–98. 10.1016/S0021-9290(01)00105-1 11672713

[48] Kotsifaki R Sideris V King E Bahr R Whiteley R. Performance and symmetry measures during vertical jump testing at return to sport after ACL reconstruction. Br J Sports Med*.* (2023) 57:1304–10. 10.1136/bjsports-2022-106588 37263763

[49] Kawakami Y Ichinose Y Fukunaga T. Architectural and functional features of human Triceps surae muscles during contraction. J Appl Physiol*.* (1998) 85:398–404. 10.1152/jappl.1998.85.2.398 9688711

[50] Saunders PU Pyne DB Telford RD Hawley JA. Factors affecting running economy in trained distance runners. Sports Med*.* (2004) 34:465–85. 10.2165/00007256-200434070-00005 15233599

[51] Liu B Wu J Shi Q Hao F Xiao W Yu J Running economy and lower extremity stiffness in endurance runners: a systematic review and meta-analysis. Front Physiol*.* (2022) 13:1059221. 10.3389/fphys.2022.1059221 36518102 PMC9742541

[52] Fletcher JR MacIntosh BR. Running economy from a muscle energetics perspective. Front Physiol*.* (2017) 8:433. 10.3389/fphys.2017.00433 28690549 PMC5479897

[53] Van Hooren B Jukic I Cox M Frenken KG Bautista I Moore IS. The relationship between running biomechanics and running economy: a systematic review and meta-analysis of observational studies. Sports Med*.* (2024) 54(5):1269–316. 10.1007/s40279-024-01997-3 38446400 PMC11127892

[54] Li F Newton RU Shi Y Sutton D Ding H. Correlation of eccentric strength, reactive strength, and leg stiffness with running economy in well-trained distance runners. J Strength Cond Res*.* (2021) 35:1491–9. 10.1519/JSC.0000000000003446 31809458

[55] Myer GD Paterno MV Ford KR Hewett TE. Neuromuscular training techniques to target deficits before return to sport after anterior cruciate ligament reconstruction. J Strength Cond Res*.* (2008) 22:987–1014. 10.1519/JSC.0b013e31816a86cd 18438211

[56] Nyland J Wera J Klein S Caborn DNM. Lower extremity neuromuscular compensations during instrumented single leg hop testing 2–10years following ACL reconstruction. Knee. (2014) 21:1191–7. 10.1016/j.knee.2014.07.017 25245550

[57] Rice DA McNair PJ. Quadriceps arthrogenic muscle inhibition: neural mechanisms and treatm ent perspectives. Semin Arthritis Rheum*.* (2010) 40:250–66. 10.1016/j.semarthrit.2009.10.001 19954822

[58] Xu D Zhou H Quan W Gusztav F Wang M Baker JS Accurately and effectively predict the ACL force: utilizing biomechanical landing pattern before and after-fatigue. Comput Methods Programs Biomed*.* (2023) 241: 107761. 10.1016/j.cmpb.2023.107761 37579552

[59] Xu D Zhou H Quan W Ma X Chon T-E Fernandez J New insights optimize landing strategies to reduce lower limb injury risk. Cyborg Bionic Syst*.* (2024) 5:0126. 10.34133/cbsystems.0126 38778877 PMC11109754

